# One Anastomosis Gastric Bypass Reconstitutes the Appropriate Profile of Serum Amino Acids in Patients with Morbid Obesity

**DOI:** 10.3390/jcm9010100

**Published:** 2019-12-31

**Authors:** Lukasz P. Halinski, Alicja Pakiet, Patrycja Jablonska, Lukasz Kaska, Monika Proczko-Stepaniak, Ewa Slominska, Tomasz Sledzinski, Adriana Mika

**Affiliations:** 1 Department of Environmental Analytics, Faculty of Chemistry, University of Gdansk, Wita Stwosza 63, 80-308 Gdansk, Poland; 2 Department of Biochemistry, Faculty of Medicine, Medical University of Gdansk, Debinki 1, 80-211 Gdansk, Poland; 3 Department of General, Endocrine and Transplant Surgery, Faculty of Medicine, Medical University of Gdansk, Smoluchowskiego 17, 80-214 Gdansk, Poland; 4 Department of Pharmaceutical Biochemistry, Medical University of Gdansk, Debinki 1, 80-211 Gdansk, Poland; tsledz@gumed.edu.pl

**Keywords:** amino acids, essential amino acids, bariatric surgery, LC-MS, principal component analysis

## Abstract

Bariatric surgery leads to metabolic benefits in patients with obesity, but their mechanisms are not well understood. The appropriate composition of serum amino acids (AA) is important for sufficient supply of these components into various tissues and organs. Obesity leads to alterations in serum AA concentrations. The aim of this study was to examine the effect of one anastomosis gastric bypass (OAGB), a promising type of bariatric surgery, on serum AA concentrations, which were assayed by LC-MS in serum of 46 bariatric patients prior to and 6–9 months after OAGB, as well as in 30 lean control subjects. The results were analyzed by principle components analysis and metabolic pathway analysis. PCA analysis showed that OAGB led to normalization of serum AA concentrations of patients with obesity to a pattern similar to the control subjects, and the concentrations of essential AA remained decreased after OAGB. Changes of individual AA and their associated metabolic pathways were also presented. OAGB caused normalization of the AA profile, which may contribute to improvement of glucose homeostasis and reduction of cardiovascular risk. Considering decreased essential AA concentrations after OAGB, increased intake of high protein food should be recommended to the patients after this type of bariatric surgery.

## 1. Introduction

One anastomosis gastric bypass (OAGB) is a minimally invasive bariatric procedure used for almost 20 years. The impact of OAGB on the metabolism of various compounds at this time has been very poorly described. Current research shows that OAGB is more effective for weight loss than Roux-en-Y gastric bypass (RYGB) and laparoscopic sleeve gastrectomy (LSG), but may negatively impact the liver, which is suggested by increased serum activity of liver enzymes, including alanine aminotransferase, aspartate aminotransferase, and alkaline phosphatase [1]. Moreover, it may lead to malnutrition [2]. However, other authors emphasized the advantages of OAGB, such as the short operative time, sustained results of excess weight loss, better remission of type 2 diabetes mellitus (T2DM) compared with RYGB, and low morbidity and mortality rates [2], as well as, most importantly, the lack of complications associated with intestine obstruction and internal herniation [3]. Some authors emphasize that OAGB is safer and simpler and can be an alternative surgery to RYGB [4]. Additionally, our earlier study showed better bile acid absorption from longer bile loops in patients after OAGB compared to RYGB and LSG, which was associated with better improvement in insulin sensitivity [5].

Alterations of the levels of essential components in each organism may be dangerous for the metabolism, function, and survival of every cell. In metabolic diseases, including obesity and type 2 diabetes mellitus (T2DM), the deficiency of some amino acids (AA), as well as the excess of others have been described [6,7,8,9,10,11,12,13]. A few years ago, AAs were considered an effective component whose concentrations were altered in T2DM and obesity [14]. Nonessential (dispensable) AAs in human organisms are synthesized de novo and are not diet dependent. Essential (indispensable) AAs are not synthesized de novo and must be obtained from the diet. However, under stress and catabolic states, some nonessential AAs become essential and are then classified as conditionally essential AAs [15]. AAs are involved in a number of important metabolic pathways [16]. Several factors may affect the serum AA concentrations, including undernutrition, catabolic stress, and the pattern and quantities of dietary AAs [7].

Decreased food uptake after bariatric surgery (BS) may lead to deficiency of some AAs, especially essential AAs. The aim of the study was to evaluate the impact of OAGB on AA concentrations in the serum of patients with morbid obesity using principal components analysis (PCA). Additionally, using pathway analysis, we presented how the metabolic pathways associated with AAs are changed after BS. Assaying the exact amino acid concentrations allows obtaining more detailed information about dietary deficits or alterations related to protein metabolism than simple albumin or total protein assays.

## 2. Experimental Section

### 2.1. Patients and Control Subjects

The study included 46 patients who qualified for surgical treatment of morbid obesity at the Department of General, Endocrine and Transplant Surgery in the period of 2016–2018. All patients enrolled in the study underwent OAGB. In each case, 180 cm of the small intestine was removed from the passage (the ascending arm of the omega loop), and a standardized 50 mL stomach pouch was created. Half of the patients from the morbidly obese group suffered from T2DM, and the other 23 had reference glucose levels. The control group consisted of 30 lean individuals without metabolic disorders. Anthropometric and laboratory parameters were determined at baseline (before surgery) and again 6–9 months after OAGB. All blood samples were collected after an overnight fast. Serum was obtained by centrifugation and stored at −80 °C. Routine laboratory parameters were determined at the Central Clinical Laboratory, Medical University of Gdansk. The anthropometric and metabolic data of patients with morbid obesity and lean controls are presented in Table 1.

The study was performed in agreement with the principles of the Declaration of Helsinki of the World Medical Association. The study protocol was approved by the Local Bioethics Committee at the Medical University of Gdansk (Decision No. NKBBN/493/2016), and written informed consent was obtained from all participants.

### 2.2. LC/MS Analysis of Amino Acids

Amino acid concentrations were determined according to Olkowicz et al. [17]. Briefly, to establish concentrations of AAs and their derivatives, an aliquot of 25 µL of serum was extracted with acetonitrile in a 1:2.4 ratio, and then, 5 µL of the internal standard solution (2-chloroadenosine) were added. Samples were agitated at 1400 rpm for 5 min, left on ice for 20 min, and centrifuged for 15 min at 14,000× *g* at 4 °C. The supernatants were collected and freeze dried. The residue was dissolved in 50 µL of water, centrifuged for 15 min at 14,000× *g* at 4 °C, and analyzed using high performance liquid chromatography-mass spectrometry (LC/MS). The analysis was conducted on a Surveyor HPLC system coupled with a TSQ Vantage Triple-Stage Quadrupole mass spectrometer (Thermo Fisher Scientific, Waltham, MA, USA). Heated electrospray ionization in positive mode was used. Chromatographic separation was achieved with a 50 × 2 mm Synergi Hydro-RP 100 column with a 2.5 µm particle size (Phenomenex, Torrance, CA, USA). The mobile phase consisted of water with 5 mM nonafluoropentanoic acid (Buffer A) and acetonitrile with 0.1% formic acid (Buffer B). Two microliter aliquots of samples were injected into a column eluted with a mobile phase at a flow rate of 0.2 mL/min. Individual amino acids and internal standards were identified, with the identity confirmed based on the similarity of molecular weights, fragmentation patterns, and chromatographic retention times.

### 2.3. Data Analysis

The data analysis was carried out using the computing environment R [18]. Principal component analysis (PCA) was performed using the FactoMineR package [19] with the factoextra package for data visualization. All data matrices were auto-scaled before the analysis. The PCA results were statistically processed using ANOVA with the Tukey–Kramer post hoc test, and differences were accepted as statistically significant at *p <* 0.01.

Pathway analysis was performed with the application of MetaboAnalyst 4.0 [20], a main tool for metabolic analysis [9] (available online: http://www.metaboanalyst.ca/).

## 3. Results

**Figure 1:**
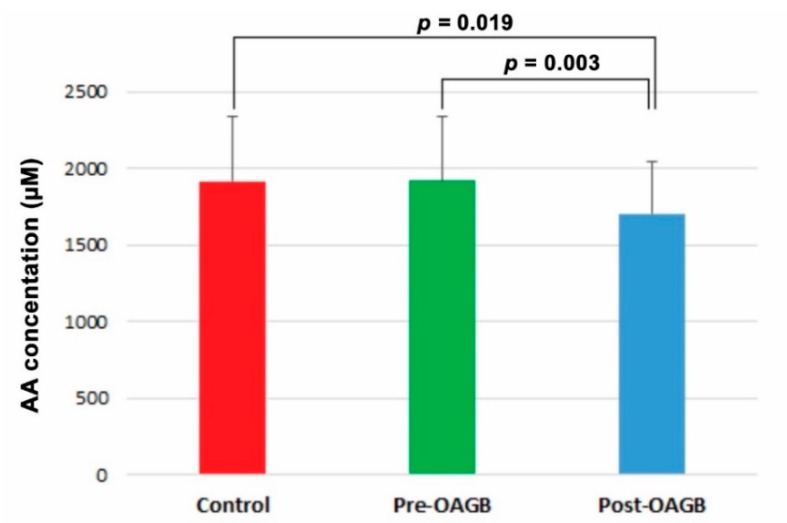
Total amino acid concentrations in the serum of patients with morbid obesity before and after OAGB and lean controls. Values are the mean ± SD.

Figure 1 presents the total concentration of AAs in the serum of patients with morbid obesity before and after OAGB and lean healthy controls (LCs). The levels of total serum AAs did not differ between LCs and obese patients before OAGB, but it decreased slightly and significantly after OAGB (Figure 1).

**Figure 2:**
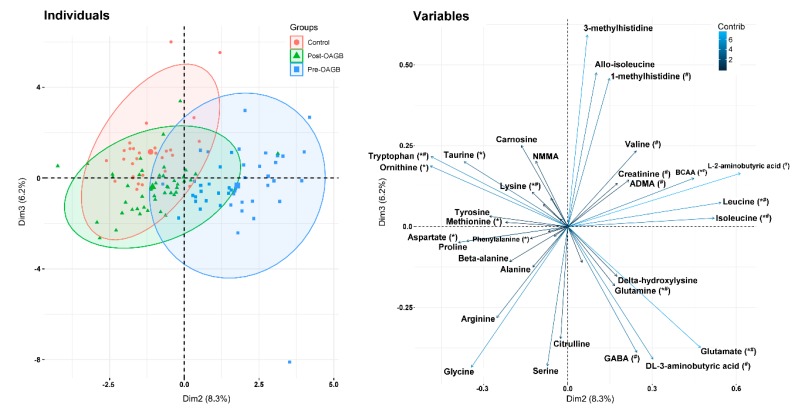
The results of principal component analysis (PCA) of individuals based on the whole amino acid profile: Score plot of cases (**left**) and variables (**right**) for the second and third PCs. Variables marked with (*) displayed statistically significant differences in their average concentrations between the control group and pre-OAGB patients, while variables marked with (#) showed such differences between patients post-OAGB and pre-OAGB. The direction of the arrows shows the correlations of variables (single compounds) with given PCs, e.g., arrows for leucine and isoleucine directed right suggest their strong positive contribution to the second PC and their higher average concentration in serum of individuals placed on the right-hand side of the score of cases (**left**). Dim, dimension; Contrib, contribution.

The principal component analysis results revealed a significant difference in the amino acid profiles among patients before OAGB and after OAGB and the LC group. The majority of variability in the dataset (PC1, 32.4%) was associated with a broad dispersion of the results within all three groups (Figure S1). It must be noted, however, that the average value of PC1 within post-OAGB patients was significantly lower than that in other groups, probably because of the lower overall plasma AA concentration (Tukey–Kramer, *p <* 0.01). In addition, PC2 was responsible for the partial separation of pre-OAGB patients from the other groups, based on the high levels of L-2-aminobutyric acid, leucine, isoleucine, and glutamic acid and low amounts of tryptophan, ornithine, taurine, aspartic acid, and proline (Figure 2). This was supported by the significantly higher average PC2 value in the pre-OAGB group when compared to the values obtained for the LC and post-OAGB groups (*p <* 0.01). Some more subtle differences in the non-essential AA profile were, on the other hand, responsible for the significantly higher PC3 value in LCs when compared to the respective values in both groups of patients. The analysis, restricted to the common amino acid profile excluding cysteine, which has not been assayed, gave similar, but clearer results. The majority of the variation in the dataset (PC1, 44.7%) was again associated with large natural variability in the amino acid profiles within all groups studied (Figure S2), and no differences in PC1 values were found between groups. Once again, PC2 (10.8% of the total variance) allowed us to separate the control group and post-OAGB patients from the pre-OAGB group (Figure S3). The latter displayed higher average levels of leucine, isoleucine, glutamate, and glutamine and lower respective values for tryptophan, glycine, and aspartate, which was supported by the significantly higher average PC2 values in pre-OAGB patients than those in the other groups. An overview of the mean values of PC1–3 obtained for each group of patients is given in Table S1. The similarity of common AA profiles in post-OAGB patients and the control group suggested the beneficial impact of the procedure on AA metabolism. Post-OAGB patients displayed a common AA profile very similar to that found in the control group (Figure S3). However, due to the important roles of AAs other than common AAs, including their application as markers of risk of various metabolic and cardiovascular diseases, we focused on data from Figure 2.

Table 2 presents the concentrations of the examined AAs detected in our study. Reduced levels of tryptophan, ornithine, lysine, glycine, taurine, aspartate, and tyrosine were detected in obese patients before OAGB compared to levels in LCs (Table 2).

In obese subjects, we observed elevated concentrations of two branched chain amino acids (BCAAs), leucine and isoleucine, as well as glutamine and glutamate (Table 2). After BS, the levels of most of the mentioned AAs showed a trend of returning to the correct concentrations similar to that observed in the LC group (Table 2). Additionally, elevated levels of L-2-aminobutyric acid (AABA) were observed before OAGB and significantly decreased after OAGB to lower levels than those in lean subjects. The second isomer of aminobutyric acid, DL-3-aminobutyric acid (BABA), was also detected at an elevated level before BS, but its level after OAGB was close to the level in the LC group. Similarly, the amounts of γ-aminobutyric acid (GABA) in obese patients significantly decreased after OAGB (Table 2).

**Figure 3:**
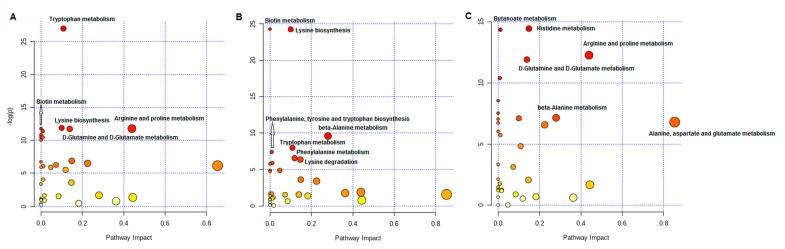
Pathway analysis for comparison of patients before OAGB with LCs (**A**), patients after OAGB with the LC group (**B**), and patients after OAGB with those before OAGB (**C**).

When considering groups of amino acids with similar properties or metabolic functions, we found increased concentrations of BCAAs, aromatic AAs, and urea cycle metabolites (Table 3). After OAGB, BCAAs and essential AAs decreased, whereas urea cycle metabolites increased (Table 3). Pathway analysis showed the most significant changes in the metabolism of tryptophan, biotin, arginine, proline, glutamine, and glutamate, as well as the biosynthesis of lysine when comparing patients before OAGB with LCs (Figure 3A). When comparing these two groups, the most significant changes among individual metabolites were found in tryptophan (*p* < 0.0001), lysine (*p* < 0.0001), ornithine (*p* < 0.0001), and glutamine (*p* < 0.0001) (Table S2). Comparison of the AA metabolism of patients after OAGB with the LC group showed the strongest changes in the metabolism of biotin, β-alanine, tryptophan, and phenylalanine; the biosynthesis of lysine, as well as phenylalanine, tyrosine, and tryptophan; and lysine degradation (Figure 3B). Among individual metabolites, lysine (*p* < 0.0001), GABA (*p* < 0.0001), tryptophan (*p =* 0.0003), and phenylalanine (*p =* 0.0001) changed the most (Table S3). In turn, butanoate, histidine, arginine, proline, glutamate, glutamine, β-alanine, alanine, aspartate, and glutamate metabolism were most significantly changed when comparing the results of patients after OAGB with those before surgery (Figure 3C). Individually, glutamate (*p* < 0.0001) and, to a lower degree, glutamine (*p =* 0.0001) metabolism was changed (Table S4).

## 4. Discussion

Obesity and being overweight have alarmingly increased worldwide during the last thirty years. Obesity is associated with many comorbidities that sometimes may lead to death. Metabolic complications include glucose intolerance and insulin resistance, metabolic syndrome, oxidative stress, and low grade inflammation [21]. Scientists are looking for an effective treatment to improve the patient’s condition and prevent comorbidities. At present, the most effective is BS. OAGB is a bariatric procedure that improves metabolic parameters [5], causes remission of type 2 diabetes mellitus [4], and is associated with low morbidity [22].

Our data indicate that OAGB improves the AA profile in obese patients, and the concentrations of AAs returned to values close to those found in serum in the LC group. This characteristic of OAGB may contribute to the improvement of the health state of obese patients because the correct level and composition of AAs in serum is important for metabolic processes in various tissues.

Some authors reported hypoaminoacidaemia during obesity [23]. However, our study did not show any significant difference in AA concentrations between LCs and obese patients before OAGB (Figure 1). In contrast, a slight, but significant decrease in total AAs in patients’ serum was observed after OAGB (Figure 1). Patients after BS are subjected to a restrictive diet, which can significantly decrease the content of the AAs supplied from the diet [7]. This supposition is particularly confirmed by the decreased levels of essential AAs after OAGB (Figure 1). Thus, increased intake of high protein food should be recommended for OAGB patients.

PCA showed significantly elevated concentrations of l-2-aminobutyric acid (AABA) in obese patients before OAGB (Figure 2). AABA is a metabolite of the catabolism of threonine [16,24], serine [25], and methionine [16]. One of the pharmaceutical applications of AABA is its use as a substrate in the synthesis of several important drugs, including the anti-epileptics brivaracetam and levetiracetam and the anti-tuberculotic ethambutol [24]. Some authors emphasized that the concentration of AABA is associated with hemodynamic changes. AABA activates AMP-kinase (AMPK) and increases intracellular glutathione levels, thus protecting against oxidative stress [25]. Oxidative stress is, in principle, significantly increased in obesity and decreases after BS [26,27], similar to the concentration of AABA (Table 2), which suggests its role in the regulation of oxidative stress associated with obesity. Additionally, the levels of DL-3-aminobutyric acid (BABA) were higher before OAGB than after OAGB. Many authors have shown beneficial properties of BABA, but only in plants. BABA is responsible for protection against abiotic and biotic stresses [28] and is involved in disease resistance and plant development [29], and its levels are elevated very quickly after biotrophic, necrotrophic, and hemibiotrophic pathogens [30]. What is interesting is that the changes in BABA in human diseases were not examined. The above information indicates the significance of this acid in plants and its potential role in human diseases. The last isomer of aminobutyric acid, γ-aminobutyric acid (GABA), also significantly decreased after OAGB (Table 2). GABA is associated with the downregulation of proinflammatory adipokines in inflammatory diseases [31], and after BS, a significant reduction in inflammation is observed [32]. Indeed, we also observed significantly reduced levels of GABA and reduced CRP after OAGB (Table 1 and Table 2). Elevated levels of AABA and BABA in obese subjects may constitute a mechanism protecting their organisms from oxidative stress, inflammation, and perhaps some other adverse conditions. After bariatric surgery, when oxidative stress and inflammation are decreased, the levels of these metabolites also decrease.

Increased levels of glutamate, glutamine, leucine, and isoleucine were observed among obese subjects compared to the LC group (Table 2). The changes in glutamine and glutamate concentrations will be discussed in the next section. Branched chain amino acids (BCAAs), including leucine, isoleucine, and valine, are associated with insulin resistance and type 2 diabetes mellitus [6,8,9,12,13,32]. Many authors emphasized the association of BCAAs with obesity [6,8,9,10,11,13]. This is also confirmed in our recent paper [33]. In our patients, BCAA concentrations decreased after OAGB (Table 2), and we observed improvements in insulin, HOMA-IR, and HbA-1C levels after BS (Table 1). Decreased aromatic AAs, and more specifically phenylalanine and tyrosine, in obese subjects result in less substrates for the synthesis of catecholamines, including adrenaline. Indeed, Reimann et al. [34] reported decreased fasting adrenaline levels in obese subjects. Since adrenaline controls metabolism in various tissues, including liver and adipose tissue, this could be involved in the development of metabolic abnormalities associated with obesity. The urea cycle occurs in the liver. Decreased concentrations of AAs that are urea cycle metabolites in obese subjects and normalization of their levels after OAGB are probably associated with deterioration of liver function in obese patients and its improvement after BS [35].

A decrease in ketogenic AA concentrations after OAGB may be associated with increased production of ketone bodies. There are no data on the effect of OAGB on ketone body levels, but rapid very low calorie diet induced weight loss caused an increase in β-hydroxybutyrate concentrations [36]. Additionally, the concentration of glucogenic AAs was decreased in our patients after OAGB, but since these AAs constituted the majority of all AAs, they were not associated solely with gluconeogenesis. Nevertheless, the concentration of glucose was significantly decreased after OAGB (Table 1).

The results of the statistical analysis coincided with the MetPA analysis (Table 2, Figure 3). Tryptophan metabolism changes the most in obesity (*p* < 0.0001, impact 0.11; Figure 3A, Table S2). Statistical analysis showed a significantly reduced concentration of tryptophan in obese patients before OAGB compared to that of lean subjects (Table 2). Tryptophan is a precursor of kynurenine, whose levels in obesity are very elevated [37,38]. This process is catalyzed by indoleamine 2,3-dioxygenase (IDO), whose activity is increased during the progression of obesity. The activity of IDO is stimulated by proinflammatory cytokines, which are elevated in obesity: interleukin-2 (IL-2), interferon-γ (IFN-γ), and tumor necrosis factor-alpha (TNF-α) [39]. Therefore, the reduced level of tryptophan may be associated with faster conversion to kynurenine, which has very important biological functions, including the regulation of the immune response [40] and dilating blood vessels during inflammation [41]. Ornithine is a precursor for proline synthesis [42]. This reaction is activated by reactive nitrogen species (RNS) that have microbicidal and proinflammatory properties that are important in immune responses. In the adipose tissue of obese subjects, the amount of macrophages increases, leading to activation of the expression of proinflammatory genes and production of proinflammatory adipokines, which in turn activate one of the nitric oxide synthase isomers, inducible nitric oxide synthase (iNOS) [43] (Lumeng 2007). Thus, increased production of RNS by iNOS [42] and enhanced conversion of ornithine to proline could be the reason for the significantly reduced ornithine in obese subjects before BS (Table 2). However, the concentration of proline is also decreased in obese patients compared with the concentration in LCs (Table 2). Another possible reason for the decreased ornithine might be its conversion into citrulline, which is slightly elevated in obese patients (Table 2). Research on diet induced obesity in mice also presented increased serum levels of citrulline [16].

The elevated concentrations of serum asymmetric dimethylarginine (ADMA) in obese patients (Table 2), which is a natural inhibitor of another isomer of NOS, endothelial nitric oxide synthase (eNOS) [44], suggest that serum NO levels may be reduced in obese patients. The study by Czumaj et al. [45] showed a close correlation between increased ADMA and BMI, as well as reduced serum NO levels [46], suggesting that decreasing levels of NO can be an early marker of the risk of cardiovascular disease in subjects with excessive weight [45]. NO is important in the regulation of blood pressure and blood flow and indirectly reduces platelet adhesion and aggregation [45]. The decreased ADMA after OAGB (Table 2) suggests that this surgical procedure can lead to decreased cardiovascular risk in patients. Some authors [9,12,14] observed elevated levels of lysine in patients before BS compared to levels in LCs; however, we observed lower levels of lysine in obese subjects and its impact on the biotin metabolism (7.55E06, impact 0.00; Table S2) and lysine metabolism (*p* < 0.0001, impact 0.10; Table S2) pathways (Figure 3A). After OAGB, the levels of lysine were even lower than before in our patients. These results are consistent with those of Nicoletti et al. [7], who detected significantly decreased concentrations of lysine after three months and even more after 12 months after RYGB [7]. Lysine is responsible for the correct action of the immune system, impacts on calcium homeostasis [47], conditions of connective tissue [48], and fatty acid metabolism [49]. It also plays a role in anemia, has an impact on the uptake of iron, and increases ferritin in blood [50]. Lysine is also a precursor of α-aminoadipic acid (AAD) formed during lysine degradation [12]. It seems that in our group of patients, catabolism of lysine is elevated; however, we did not investigate the AAD levels. 

The concentrations of arginine and glycine, which are precursors of creatinine, were elevated after surgery, so the decrease of serum creatinine level is rather an effect of improvement of kidney function that is observed after bariatric surgery [51].

Pathway analysis showed the most significant changes in glutamate metabolic pathways between the patients before and after OAGB (Table S4). Glutamate is involved in butanoate metabolism, histidine metabolism, arginine, and proline metabolism, as well as in D-glutamine and D-glutamate metabolism (Figure 3C, Table S4). A high concentration of glutamate may increase GABA production [32]. Indeed, in obese patients before BS, we found increased glutamate and GABA concentrations (Table 2). In vitro experiments suggested that elevated intracellular glutamate is associated with adipocyte dysfunction, including altered insulin mediated glucose uptake and decreased adiponectin secretion [52]. Our study showed changes in glutamine metabolism and alterations in arginine and proline metabolism (*p* < 0.0001, impact 0.44; Table S4). Similar to glutamate, glutamine becomes an essential AA under oxidative stress, disease conditions, or catabolic states [15] and plays a role in intestinal mucosal integrity, antioxidative responses, and immunity and cell signaling [53]. Glutamine concentrations and the glutamine to glutamate ratio in plasma are strongly inversely associated with insulin resistance. Thus, a high ratio of glutamine to glutamate seems to be associated with a lower risk of T2DM [54]. In our study, the ratio of glutamine to glutamate in patients after OAGB was significantly elevated compared to that of patients before OAGB. This result suggested that the decreased glutamine to glutamate ratio may contribute to the improvement in insulin resistance observed after OAGB. The highest change in glutamine was noted in the pathway analysis of arginine and proline metabolism (*p =* 0.0001, Table S4). Ren et al. [55] described studies showing decreased levels of glutamine in the serum of patients with obesity and T2DM, which was associated with elevated accumulation of M1 macrophages and their proinflammatory properties. In our study, we also observed an elevated concentration of glutamine in obese subjects before OAGB (Table 2). In turn, Laferrere et al. [6] and Yao et al. [13] noted a reduced level of glutamine after BS, which is also consistent with our results.

When comparing different types of bariatric surgery, six months after RYGB, Wijayatunga et al. [56] found decreased BCAA and increased glycine, which was consistent with our results in patients after OAGB. The difference between our and Wijayatunga et al.’s [56] results was that they found also increased serum alanine and taurine, whereas we did not find significant differences in these metabolites. However, it should be noted that their methodology was different. In turn, six months after sleeve gastrectomy, Yao et al. [13] found decreased serum BCAA, glutamate, and glutamine similar to our patients, but their patients had additionally decreased alanine, methionine, phenylalanine, and tyrosine after BS, which were not changed in our patients after OAGB.

It should be also mentioned that in the study by Robert et al. [57], at two year follow-up in patients after OAGB, among the serious events associated with surgery, 21% were nutritional complications, including vitamin and iron deficiency or malnutrition. This may affect the concentrations of amino acids in some patients.

The relatively small cohort was a limitation of our study, but even with this sample size, the statistical significance of the results was quite robust and therefore convincing.

## 5. Conclusions

The reduction in food intake and malabsorption after OAGB induced weight loss and modified serum concentrations of amino acids. Our PCA analysis clearly indicated a change in the serum amino acid concentrations after OAGB to values close to the control group. These post-OAGB changes in amino acid concentrations seemed to be beneficial for patients’ health, contributing to the improvement in glucose homeostasis and reduction in cardiovascular risk. However, considering the decreased essential AA concentrations after OAGB, increased intake of high protein food should be recommended to patients after this type of BS.

## Figures and Tables

**Table 1:** Metabolic characteristics of patients with morbid obesity before and after bariatric surgery and lean controls. Values are the mean ± SD. Abbreviations: LCs: Lean controls, OAGB: One anastomosis gastric bypass; BMI: Body mass index; TC: Total cholesterol, CRP: C-reactive protein, HOMA-IR: Homeostatic model assessment-insulin resistance, TG: Triglyceride, HDL: High density lipoprotein, LDL: Low density lipoprotein.

	Lean Controls (LC)	Pre-OAGB	6–9 m Post-OAGB	*p* (Pre-OAGB vs. LC)	*p* (Pre- vs. Post-OAGB)	*p* (Post-OAGB vs. LC)
Age (years)	49.71 ± 11.25	48.60 ± 10.57		0.685	-	0.685
BMI (kg/m^2^)	24.9 ± 2.57	38.5 ± 4.31	29.6 ± 3.85	<0.001	<0.001	<0.001
HbA1C (%)	-	5.79 ± 0.88	5.24 ± 0.47	-	0.003	-
TG (mg/dL)	109 ± 47.7	113 ± 37.3	87.8 ± 26.7	0.772	0.006	0.080
HDL (mg/dL)	55.3 ± 13.2	50.1 ± 9.33	50.9 ± 11.79	0.125	0.653	0.273
LDL (mg/dL)	128.4 ± 41.7	114 ± 33.8	88.3 ± 25.3	0.241	0.027	<0.001
TC (mg/dL)	207.8 ± 44.5	201 ± 40.9	180 ± 49.9	0.486	0.018	0.039
CRP (mg/L)	1.57 ± 1.22	3.24 ± 4.94	1.02 ± 0.55	0.217	0.010	0.075
Albumin (g/L)	40.0 ± 2.34	37.4 ± 7.60	37.1 ± 2.45	0.135	0.832	<0.001
Creatinine (mg/dL)	0.86 ± 0.16	0.80 ± 0.22	0.71 ± 0.17	0.246	<0.001	<0.001
Glucose (mg/dL)	93.1 ± 9.36	111 ± 32.1	91.5 ± 11.2	0.004	<0.001	0.468
Insulin (µU/mL)	9.11 ± 3.97	14.9 ± 7.85	7.72 ± 6.49	0.002	<0.001	0.335
HOMA-IR	2.13 ± 1.02	4.37 ± 3.04	2.04 ± 1.96	0.001	<0.001	0.804

**Table 2:** Concentrations of amino acids (AAs) (µM) detected in serum of patients with morbid obesity and lean subjects. Values are the mean ± SD.

AA	LC	Pre-OAGB	Post-OAGB	*p* (Pre-OAGB vs. LC)	*p* (Pre- vs. Post-OAGB)	*p* (Post-OAGB vs. LC)
Alanine	286 ± 107	274 ± 109	261 ± 78.7	0.638	0.486	0.247
Arginine	50.1 ± 15.0	45.6 ± 12.5	49.3 ± 12.0	0.167	0.108	0.794
Asparagine	16.7 ± 5.68	16.4 ± 6.03	14.0 ± 4.63	0.818	0.022	0.027
Aspartic acid	13.3 ± 4.41	9.52 ± 4.98	11.8 ± 5.08	0.001	0.042	0.172
Glutamate	34.4 ± 20.7	66.8 ± 50.0	24.7 ± 16.3	0.001	<0.001	0.026
Glutamine	363 ± 84.2	446 ± 72.0	386 ± 77.8	<0.001	<0.001	0.241
Glycine	159 ± 51.0	146 ± 70.2	162 ± 75.1	<0.001	<0.001	0.241
Histidine	48.0 ± 10.9	44.1 ± 8.67	41.7 ± 10.5	0.090	0.180	0.015
Isoleucine	32.0 ± 12.7	45.9 ± 14.5	28.4 ± 10.5	<0.001	<0.001	0.195
Leucine	57.6 ± 19.2	77.1 ± 20.0	47.4 ± 18.3	<0.001	<0.001	0.024
Lysine	143 ± 30.7	111 ± 25.7	93.2 ± 23.7	<0.001	<0.001	<0.001
Methionine	13.8 ± 3.78	11.5 ± 3.92	10.3 ± 3.75	0.013	0.119	<0.001
Phenylalanine	38.5 ± 9.67	32.9 ± 11.6	30.6 ± 7.46	0.033	0.209	<0.001
Proline	94.3 ± 33.1	83.2 ± 22.2	99.9 ± 38.0	0.085	0.001	0.517
Serine	75.3 ± 18.1	79.9 ± 22.8	74.4 ± 20.9	0.366	0.170	0.840
Threonine	48.4 ± 14.8	49.3 ± 15.2	37.6 ± 18.3	0.802	0.001	0.009
Tryptophan	28.1 ± 7.20	16.3 ± 4.90	20.5 ± 9.30	<0.001	0.006	<0.001
Tyrosine	45.8 ± 13.5	36.9 ± 11.0	36.8 ± 14.0	0.002	0.964	0.007
Valine	128 ± 34.8	123 ± 35.9	97.7 ± 25.5	0.417	<0.001	<0.001
Betaine	25.9 ± 5.61	24.7 ± 7.94	24.3 ± 6.93	0.504	0.709	0.324
Carnosine	0.10 ± 0.11	0.07 ± 0.06	0.08 ± 0.09	0.087	0.360	0.366
Citrulline	3.56 ± 1.50	4.12 ± 1.67	3.80 ± 1.64	0.146	0.341	0.525
Creatinine	45.6 ± 14.0	46.7 ± 15.7	36.8 ± 12.0	0.773	<0.001	0.004
Ornithine	47.3 ± 16.9	31.1 ± 11.3	35.1 ± 13.8	<0.001	0.067	0.001
Taurine	49.0 ± 23.6	33.2 ± 17.1	32.3 ± 18.2	0.001	0.807	<0.001
DL-3-aminobutyric acid (BABA)	0.29 ± 0.25	0.73 ± 0.84	0.35 ± 0.38	0.008	0.008	0.476
γ-aminobutyric acid (GABA)	25.8 ± 5.97	26.2 ± 7.65	17.2 ± 4.23	0.814	<0.001	<0.001
L-2-aminobutyric acid (AABA)	8.46 ± 3.74	10.9 ± 4.33	3.11 ± 2.51	0.014	<0.001	<0.001
1-methyl-histidine	2.23 ± 0.87	1.99 ± 0.96	1.54 ± 0.97	0.269	0.001	0.002
3-methyl-histidine	23.8 ± 31.9	15.2 ± 14.7	13.2 ± 24.6	0.119	0.619	0.110
β-alanine	9.19 ± 3.21	8.27 ± 3.08	8.76 ± 2.55	0.216	0.380	0.515
Asymmetric dimethylarginine (ADMA)	0.16 ± 0.11	0.21 ± 0.14	0.14 ± 0.09	0.117	0.003	0.303
Mono-L-methylarginine (NMMA)	0.09 ± 0.07	0.06 ± 0.05	0.06 ± 0.05	0.036	0.910	0.036
Symmetric dimethylarginine (SDMA)	0.24 ± 0.13	0.21 ± 0.12	0.17 ± 0.12	0.282	0.135	0.020
Glutamine/glutamate ratio	17.07 ± 15.04	10.53 ± 8.19	24.66 ± 19.73	0.017	<0.001	0.081

**Table 3:** Serum concentrations of various groups of AAs (µM). Values are the mean ± SD. BCAA, branched chain AA.

AA	LC	Pre-OAGB	Post-OAGB	*p* (Pre-OAGB vs. LC)	*p* (Pre- vs. Post-OAGB)	*p* (Post-OAGB vs. LC)
BCAA ^1^	218 ± 60	246 ± 54	174 ± 46	0.045	<0.001	0.001
Essential AA ^2^	489 ± 107	466 ± 93	366 ± 87	0.337	<0.001	<0.001
Non-essential AA ^3^	1186 ± 269	1249 ± 291	1161 ± 242	0.359	0.086	0.683
Aromatic AA ^4^	112 ± 27	86 ± 23	88 ± 27	<0.001	0.704	<0.001
Glucogenic AA ^5^	1475 ± 332	1527 ± 335	1386 ± 285	0.519	0.019	0.228
Ketogenic AA ^6^	344 ± 73	320 ± 69	257 ± 60	0.151	<0.001	<0.001
Urea cycle metabolites ^7^	114 ± 28	90 ± 24	100 ± 22	<0.001	0.037	0.019

^1^ BCAA: leucine, isoleucine, valine; ^2^ essential AA: Tryptophan, leucine, isoleucine, lysine, phenylalanine, methionine, valine, threonine; ^3^ non-essential: glutamate, glutamine, aspartate, asparagine, alanine, glycine, tyrosine, arginine, histidine, proline, serine; ^4^ aromatic AA: tyrosine, phenylalanine, tryptophan; ^5^ glucogenic AA: Tryptophan, isoleucine, phenylalanine, methionine, valine, threonine, glutamate, glutamine, aspartate, asparagine, alanine, glycine, tyrosine, arginine, histidine, proline, serine; ^6^ ketogenic AA: Leucine, lysine, phenylalanine, isoleucine, threonine, tryptophan, tyrosine; ^7^ urea cycle metabolites: Citrulline, ornithine, aspartate, arginine.

## Supplementary Materials

The following are available online at https://www.mdpi.com/2077-0383/9/1/100/s1: Figure S1: The results of principal component analysis (PCA) of individuals based on the whole amino acid profile: score plot of cases for the first two PCs, Figure S2: The results of principal component analysis (PCA) of individuals based on the essential amino acid profile: score plot of cases for the first two PCs, Figure S3: The results of principal component analysis (PCA) of individuals based on the common amino acid profile: score plot of cases (left) and variables (right) for the second and third PC, Table S1: Mean values of first three principal components from PCA analysis based on the AA profiles, Table S2. The most important impact of pathway analysis with MetPA from serum endogenous metabolites. Comparison of metabolites from serum of lean controls with pre-OAGB patients, Table S3. The most important impact of pathway analysis with MetPA from serum endogenous metabolites. Comparison of metabolites from serum of lean controls with post-OAGB patients, Table S4. The most important impact of pathway analysis with MetPA from serum endogenous metabolites. Comparison of metabolites from serum of pre-OAGB with post-OAGB patients.

## References

[1] Spivak H., Munz Y., Rubin M., Raz I., Shohat T., Blumenfeld O. (2018). Omega-loop gastric bypass is more effective for weight loss but negatively impacts liver enzymes: A registry-based comprehensive first-year analysis. Surg. Obes. Relat. Dis..

[2] Lee W.J., Lin Y.H. (2014). Single-Anastomosis Gastric Bypass (SAGB): Appraisal of Clinical Evidence. Obes. Surg..

[3] Lee W.J., Ser K.H., Lee Y.C., Tsou J.J., Chen S.C., Chen J.C. (2012). Laparoscopic Roux-en-Y Vs. Mini-gastric Bypass for the Treatment of Morbid Obesity: A 10-Year Experience. Obes. Surg..

[4] Mahawar K.K., Jennings N., Brown J., Gupta A., Balupuri S., Small P.K. (2013). “mini” gastric bypass: Systematic review of a controversial procedure. Obes. Surg..

[5] Mika A., Kaska L., Proczko-Stepaniak M., Chomiczewska A., Swierczynski J., Smolenski R.T., Sledzinski T. (2018). Evidence That the Length of Bile Loop Determines Serum Bile Acid Concentration and Glycemic Control After Bariatric Surgery. Obes. Surg..

[6] Laferrere B., Reilly D., Arias S., Swerdlow N., Gorroochurn P., Bawa B., Bose M., Teixeira J., Stevens R.D., Wenner B.R. (2011). Differential Metabolic Impact of Gastric Bypass Surgery Versus Dietary Intervention in Obese Diabetic Subjects Despite Identical Weight Loss. Sci. Transl. Med..

[7] Ferreira Nicoletti C., Morandi Junqueira-Franco M.V., Dos Santos J.E., Sergio Marchini J., Junior W.S., Nonino C.B. (2013). Protein and amino acid status before and after bariatric surgery: A 12-month follow-up study. Surg. Obes. Relat. Dis..

[8] Rauschert S., Uhl O., Koletzko B., Hellmuth C. (2014). Metabolomic Biomarkers for Obesity in Humans: A Short Review. Ann. Nutr. Metab..

[9] Samczuk P., Ciborowski M., Kretowski A. (2018). Application of Metabolomics to Study Effects of Bariatric Surgery. J. Diabetes Res..

[10] Lopes T.I.B., Geloneze B., Pareja J.C., Calixto A.R., Ferreira M.M.C., Marsaioli A.J. (2015). Blood Metabolome Changes Before and After Bariatric Surgery: A ^1^ H NMR-Based Clinical Investigation. Omi. A J. Integr. Biol..

[11] Lerin C., Goldfine A.B., Boes T., Liu M., Kasif S., Dreyfuss J.M., De Sousa-Coelho A.L., Daher G., Manoli I., Sysol J.R. (2016). Defects in muscle branched-chain amino acid oxidation contribute to impaired lipid metabolism. Mol. Metab..

[12] Libert D.M., Nowacki A.S., Natowicz M.R. (2018). Metabolomic analysis of obesity, metabolic syndrome, and type 2 diabetes: Amino acid and acylcarnitine levels change along a spectrum of metabolic wellness. PeerJ.

[13] Yao J., Kovalik J.P., Lai O.F., Lee P.C., Eng A., Chan W.H., Tham K.W., Lim E., Bee Y.M., Tan H.C. (2018). Comprehensive Assessment of the Effects of Sleeve Gastrectomy on Glucose, Lipid, and Amino Acid Metabolism in Asian Individuals with Morbid Obesity. Obes. Surg..

[14] Takashina C., Tsujino I., Watanabe T., Sakaue S., Ikeda D., Yamada A., Sato T., Ohira H., Otsuka Y., Oyama-Manabe N. (2016). Associations among the plasma amino acid profile, obesity, and glucose metabolism in Japanese adults with normal glucose tolerance. Nutr. Metab..

[15] Morris C.R., Hamilton-Reeves J., Martindale R.G., Sarav M., Ochoa Gautier J.B. (2017). Acquired Amino Acid Deficiencies: A Focus on Arginine and Glutamine. Nutr. Clin. Pract..

[16] Sailer M., Dahlhoff C., Giesbertz P., Eidens M.K., de Wit N., Rubio-Aliaga I., Boekschoten M.V., Müller M., Daniel H. (2013). Increased Plasma Citrulline in Mice Marks Diet-Induced Obesity and May Predict the Development of the Metabolic Syndrome. PLoS ONE.

[17] Olkowicz M., Debski J., Jablonska P., Dadlez M., Smolenski R.T. (2017). Application of a new procedure for liquid chromatography/mass spectrometry profiling of plasma amino acid-related metabolites and untargeted shotgun proteomics to identify mechanisms and biomarkers of calcific aortic stenosis. J. Chromatogr. A.

[18] R Core Team (2019). R 2019: A Language and Environment for Statistical Computering.

[19] Lê S., Josse J., Husson F. (2008). FactoMineR: An *R* Package for Multivariate Analysis. J. Stat. Softw..

[20] Xia J., Wishart D.S. (2011). Web-based inference of biological patterns, functions and pathways from metabolomic data using MetaboAnalyst. Nat. Protoc..

[21] Hernández Bautista R.J., Mahmoud A.M., Königsberg M., López Díaz Guerrero N.E. (2019). Obesity: Pathophysiology, monosodium glutamate-induced model and anti-obesity medicinal plants. Biomed. Pharmacother..

[22] Rutledge R. (2001). The Mini-Gastric Bypass: Experience with the First 1,274 Cases. Obes. Surg..

[23] Jeevanandam M., Ramias L., Schiller W.R. (1991). Altered plasma free amino acid levels in obese traumatized man. Metabolism.

[24] Tao R., Jiang Y., Zhu F., Yang S. (2014). A one-pot system for production of l-2-aminobutyric acid from l-threonine by l-threonine deaminase and a NADH-regeneration system based on l-leucine dehydrogenase and formate dehydrogenase. Biotechnol. Lett..

[25] Irino Y., Toh R., Nagao M., Mori T., Honjo T., Shinohara M., Tsuda S., Nakajima H., Satomi-Kobayashi S., Shinke T. (2016). 2-Aminobutyric acid modulates glutathione homeostasis in the myocardium. Sci. Rep..

[26] Sledzinski T., Goyke E., Smolenski R.T., Sledzinski Z., Swierczynski J. (2009). Decrease in serum protein carbonyl groups concentration and maintained hyperhomocysteinemia in patients undergoing bariatric surgery. Obes. Surg..

[27] Horn R.C., Gelatti G.T., Mori N.C., Tissiani A.C., Mayer M.S., Pereira E.A., Ross M., Moreira P.R., Bortolotto J.W., Felippin T. (2017). Obesity, bariatric surgery and oxidative stress. Rev. Assoc. Med. Bras..

[28] Ton J., Jakab G. (2007). Dissecting the β -aminobutyric acid—induced priming phenomenon in Arabidopsis. Plant J..

[29] Kim Y.C., Kim Y.H., Lee Y.H., Lee S.W., Chae Y.S., Kang H.K., Yun B.W., Hong J.K. (2013). Β-Amino-N-Butyric Acid Regulates Seedling Growth and Disease Resistance of Kimchi Cabbage. Plant Pathol. J..

[30] Thevenet D., Pastor V., Baccelli I., Balmer A., Vallat A., Neier R., Glauser G., Mauch-Mani B. (2017). The priming molecule β-aminobutyric acid is naturally present in plants and is induced by stress. New Phytol..

[31] Aggarwal S., Ahuja V., Paul J. (2017). Attenuated GABAergic Signaling in Intestinal Epithelium Contributes to Pathogenesis of Ulcerative Colitis. Dig. Dis. Sci..

[32] Aron-Wisnewsky J., Prifti E., Belda E., Ichou F., Kayser B.D., Dao M.C., Verger E.O., Hedjazi L., Bouillot J.L., Chevallier J.M. (2019). Major microbiota dysbiosis in severe obesity: Fate after bariatric surgery. Gut.

[33] Pakiet A., Wilczynski M., Rostkowska O., Korczynska J., Jabłonska P., Kaska L., Proczko-Stepaniak M., Sobczak E., Stepnowski P., Magkos F. (2019). The Effect of One Anastomosis Gastric Bypass on Branched-Chain Fatty Acid and Branched-Chain Amino Acid Metabolism in Subjects with Morbid Obesity. Obes. Surg..

[34] Reimann M., Qin N., Gruber M., Bornstein S.R., Kirschbaum C., Ziemssen T., Eisenhofer G. (2017). Adrenal medullary dysfunction as a feature of obesity. Int. J. Obes..

[35] Swierczynski J., Sledzinski T., Slominska E., Smolenski R., Sledzinski Z. (2009). Serum Phenylalanine Concentration as a Marker of Liver Function in Obese Patients Before and After Bariatric Surgery. Obes. Surg..

[36] Alemán J.O., Iyengar N.M., Walker J.M., Milne G.L., Da Rosa J.C., Liang Y., Giri D.D., Zhou X.K., Pollak M.N., Hudis C.A. (2017). Effects of Rapid Weight Loss on Systemic and Adipose Tissue Inflammation and Metabolism in Obese Postmenopausal Women. J. Endocr. Soc..

[37] Mallmann N.H., Lima E.S., Lalwani P. (2018). Dysregulation of Tryptophan Catabolism in Metabolic Syndrome. Metab. Syndr. Relat. Disord..

[38] Mangge H., Summers K.L., Meinitzer A., Zelzer S., Almer G., Prassl R., Schnedl W.J., Reininghaus E., Paulmichl K., Weghuber D. (2014). Obesity-related dysregulation of the Tryptophan-Kynurenine metabolism: Role of age and parameters of the metabolic syndrome. Obesity.

[39] Oxenkrug G.F. (2010). Metabolic syndrome, age-associated neuroendocrine disorders, and dysregulation of tryptophan-kynurenine metabolism. Ann. N. Y. Acad. Sci..

[40] Moffett J.R., Namboodiri M.A. (2003). Tryptophan and the immune response. Immunol. Cell Biol..

[41] Wang Y., Liu H., McKenzie G., Witting P.K., Stasch J.P., Hahn M., Changsirivathanathamrong D., Wu B.J., Ball H.J., Thomas S.R. (2010). Kynurenine is an endothelium-derived relaxing factor produced during inflammation. Nat. Med..

[42] Bronte V., Zanovello P. (2005). Regulation of immune responses by L-arginine metabolism. Nat. Rev. Immunol..

[43] Lumeng C.N., Bodzin J.L., Saltiel A.R., Lumeng C.N., Bodzin J.L., Saltiel A.R. (2007). Obesity induces a phenotypic switch in adipose tissue macrophage polarization Find the latest version: Obesity induces a phenotypic switch in adipose tissue macrophage polarization. J. Clin. Investig..

[44] Konukoglu D., Uzun H., Firtina S., Arica P.Ç., Kocael A., Taskin M. (2007). Plasma Adhesion and Inflammation Markers: Asymmetrical Dimethyl-L-Arginine and Secretory Phospholipase A2 Concentrations before and after Laparoscopic Gastric Banding in Morbidly Obese Patients. Obes. Surg..

[45] Czumaj A., Śledzińska M., Brzeziński M., Szlagatys-Sidorkiewicz A., Słomińska E., Śledziński T. (2019). Decreased serum level of nitric oxide in children with excessive body weight. Adv. Clin. Exp. Med..

[46] de Giorgis T., Marcovecchio M.L., Giannini C., Chiavaroli V., Chiarelli F., Mohn A. (2016). Blood pressure from childhood to adolescence in obese youths in relation to insulin resistance and asymmetric dimethylarginine. J. Endocrinol. Investig..

[47] Shoulders M.D., Raines R.T. (2009). Collagen Structure and Stability. Annu. Rev. Biochem..

[48] Civitelli R., Villareal D.T., Agnusdei D., Nardi P., Avioli L.V., Gennari C. (1992). Dietary L-lysine and calcium metabolism in humans. Nutrition.

[49] Vaz F.M., Wanders R.J.A. (2002). Carnitine biosynthesis in mammals. Biochem. J..

[50] Rushton D.H. (2002). Nutritional factors and hair loss. Clin. Exp. Dermatol..

[51] Śledziński T., Proczko-Markuszewska M., Kaska Ł., Stefaniak T., Świerczyński J. (2012). Serum Cystatin C in Relation to Fat Mass Loss After Bariatric Surgery. Pol. J. Surg..

[52] Nagao H., Nishizawa H., Bamba T., Nakayama Y., Isozumi N., Nagamori S., Kanai Y., Tanaka Y., Kita S., Fukuda S. (2017). Increased dynamics of tricarboxylic acid cycle and glutamate synthesis in obese adipose tissue: In vivo metabolic turnover analysis. J. Biol. Chem..

[53] Wu G. (2010). Functional Amino Acids in Growth, Reproduction, and Health. Adv. Nutr..

[54] Cheng S., Rhee E.P., Larson M.G., Lewis G.D., McCabe E.L., Shen D., Palma M.J., Roberts L.D., Dejam A., Souza A.L. (2012). Metabolite Profiling Identifies Pathways Associated With Metabolic Risk in Humans. Circulation.

[55] Ren W., Xia Y., Chen S., Wu G., Bazer F.W., Zhou B., Tan B., Zhu G., Deng J., Yin Y. (2019). Glutamine Metabolism in Macrophages: A Novel Target for Obesity/Type 2 Diabetes. Adv. Nutr..

[56] Wijayatunga N.N., Sams V.G., Dawson J.A., Mancini M.L., Mancini G.J., Moustaid-Moussa N. (2018). Roux-en-Y gastric bypass surgery alters serum metabolites and fatty acids in patients with morbid obesity. Diabetes Metab. Res. Rev..

[57] Robert M., Espalieu P., Pelascini E., Caiazzo R., Sterkers A., Khamphommala L., Poghosyan T., Chevallier J.M., Malherbe V., Chouillard E. (2019). Efficacy and safety of one anastomosis gastric bypass versus Roux-en-Y gastric bypass for obesity (YOMEGA): A multicentre, randomised, open-label, non-inferiority trial. Lancet.

