# Supplementary material for: One Anastomosis Gastric Bypass Reconstitutes the Appropriate Profile of Serum Amino Acids in Patients with Morbid Obesity
Source: J Clin Med. 2019 Dec 31;9(1):100. doi: 10.3390/jcm9010100 (PMC7020034; doi:10.3390/jcm9010100)
Supplement: Supplementary file 1 [file jcm-09-00100-s001.pdf]

One anastomosis gastric bypass reconstitutes the appropriate profile of serum amino acids in patients with morbid obesity

Lukasz P. Halinski [1], Alicja Pakiet [1], Patrycja Jablonska [2], Lukasz Kaska [3], Monika Proczko-Stepaniak [3], Ewa Slominska [2], Tomasz Sledzinski [4] and Adriana Mika [1,4,*]

1 Department of Environmental Analysis, Faculty of Chemistry, University of Gdansk, Wita Stwosza 63, 80-308 Gdansk, Poland; lukasz.halinski@ug.edu.pl (L.H.), alicja.pakiet@phdstud.ug.edu.pl (A.P.)
2 Department of Biochemistry, Faculty of Medicine, Medical University of Gdansk, Debinki 1, 80-211 Gdansk, Poland; patrycja.jablonska@gumed.edu.pl (P.J.), ewa.slominska@gumed.edu.pl (E.S.)
3 Department of General, Endocrine and Transplant Surgery, Faculty of Medicine, Medical University of Gdansk, Smoluchowskiego 17, 80-214 Gdansk, Poland; lukasz.kaska@gumed.edu.pl (L.K.), monika.proczko-stepaniak@gumed.edu.pl (M.P.-S.)
4 Department of Pharmaceutical Biochemistry, Medical University of Gdansk, Debinki 1, 80-211 Gdansk, Poland; tsledz@gumed.edu.pl
* Correspondence: adrianamika@tlen.pl; Tel.: +48-58-523-51-90

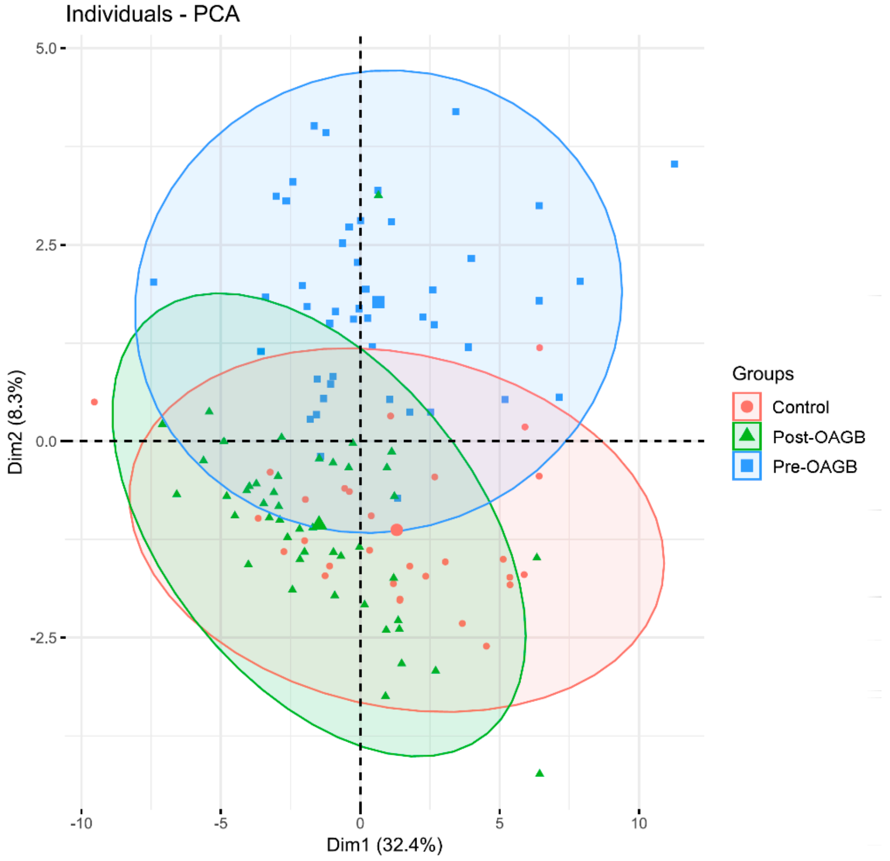

Figure S1. The results of principal component analysis (PCA) of individuals based on the whole amino acid profile: score plot of cases for first two PCs.

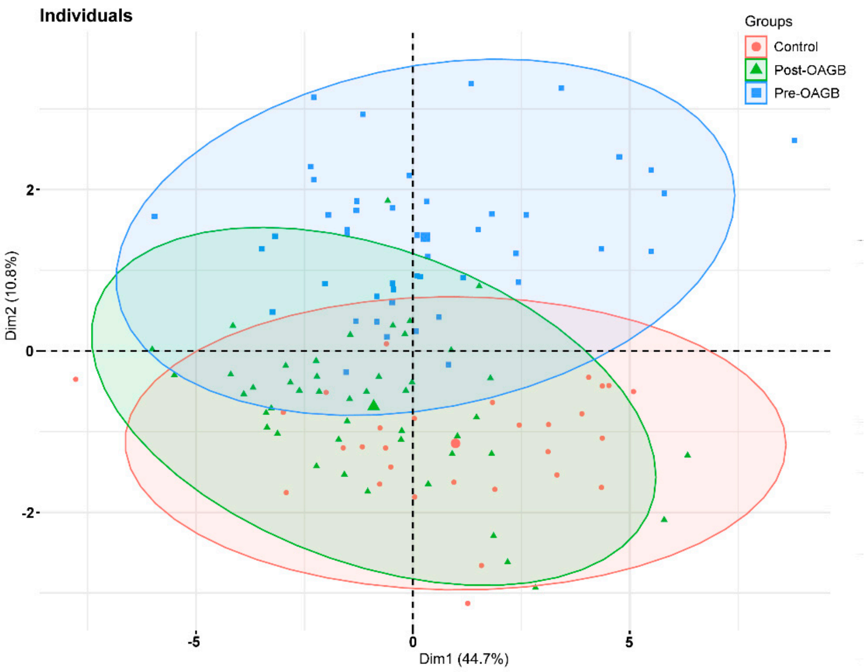

Figure S2. The results of principal component analysis (PCA) of individuals based on the essential amino acid profile: score plot of cases for first two PCs.

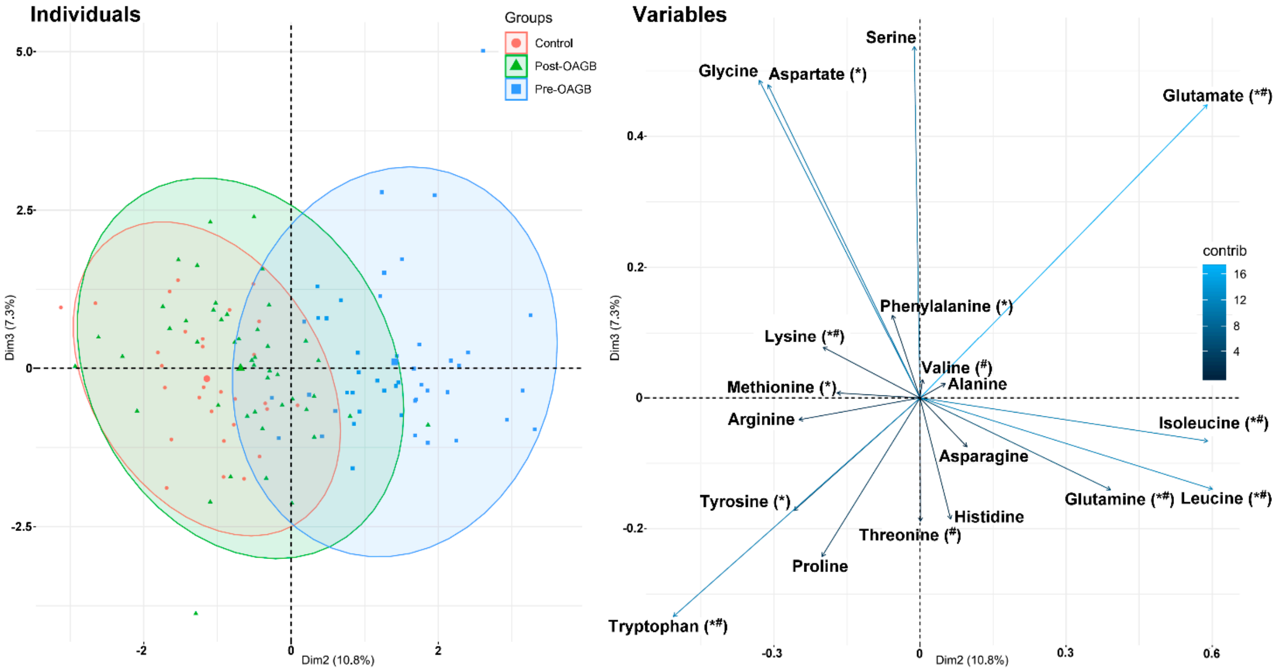

Figure S3. The results of principal component analysis (PCA) of individuals based on the common amino acid profile: score plot of cases (left) and variables (right) for the 2nd and 3rd PC. Statistical significance was marked as described in Figure 2 (main document).

Table S1. Mean values of first three principal components from PCA analysis based on the AA profiles. Values from the same analysis not sharing the same letter are significantly different from each other (Tukey-Kramer, p < 0.01).

PC no.	Whole AA profile			Common AA profile		
	LC	Pre-OAGB	Post-OAGB	LC	Pre-OAGB	Post-OAGB
1	1.31 ± 3.71a	0.65 ± 3.45a	-1.48 ± 2.93b	0.98 ± 2.95a	0.29 ± 2.82a	-0.91 ± 2.57a
2	-1.13 ± 0.90a	1.77 ± 1.16b	-1.06 ± 1.16a	-1.14 ± 0.70a	1.41 ± 0.87b	-0.69 ± 0.88a
3	1.15 ± 1.50a	-0.33 ± 1.61b	-0.39 ± 1.06b	-0.16 ± 0.96a	0.10 ± 1.22a	0.01 ± 1.19a

Table S2. The most important impact of pathway analysis with MetPA from serum endogenous metabolites. Comparison metabolites from serum of lean controls with pre-OAGB patients.

	Total Cmpd	Hits	Raw p	-log(p)	Holm adjust	FDR	Impact	Pivotal amino acid	P value
Tryptophan metabolism	79	1	1.92E-12	2.70E+01	6.72E-11	6.73E-11	0.11	L-Tryptophan	1.92E-12
Lysine biosynthesis	32	2	6.92E-06	1.19E+01	2.35E-04	5.63E-05	0.10	L-lysine	7.55E-06
Biotin metabolism	11	1	7.55E-06	1.18E+01	2.49E-04	5.63E-05	0.00	L-Lysine	7.55E-06
Arginine and proline metabolism	77	9	7.69E-06	1.18E+0.1	2.49E-04	5.63E-05	0.44	L-Glutamine; Ornithine	1.89E-05; 4.29E-06
D-Glutamine and D-glutamate metabolism	11	2	8.04E-06	1.17E+01	2.49E-04	5.63E-05	0.14	L-Glutamine	1.98E-05
Nitrogen metabolism	39	11	1.13E-05	1.14E+01	3.39E-04	6.58E-05	0.01	L-Tryptophan	1.92E-12
Pyrimidine metabolism	60	2	1.99E-05	1.08E+01	5.76E-04	9.93E-05	0.00		
D-Arginine and D-ornithine metabolism	8	2	2.83E-05	1.05E+01	7.92E-04	1.12E-04	0.00		
Phenylalanine. tyrosine and tryptophan biosynthesis	27	3	2.87E-05	1.05E+01	7.92E-04	1.12E-04	0.01		

Purine metabolism	92	2	4.41E-05	1.00E+01	1.15E-03	1.54E-04	0.00
Histidine metabolism	44	5	1.04E-03	6.87E+00	2.60E-02	3.30E-03	0.15
Nicotinate and nicotinamide metabolism	44	1	1.19E-03	6.73E+00	2.86E-02	3.47E-03	0.00
Aminoacyl t-RNA biosynthesis	75	16	1.51E-03	6.50E+00	3.47E-02	4.06E-03	0.23
Pantothenate and CoA biosynthesis	27	2	1.92E-03	6.26E+00	4.22E-02	4.80E-03	0.07
Alanine. aspartate and glutamate metabolism	24	6	2.14E-03	6.14E+00	4.50E-02	5.00E-03	0.85
Butanoate metabolism	40	2	2.32E-03	6.07E+00	4.62E-02	5.05E-03	0.01
Tyrosine metabolism	76	1	2.74E-03	5.90E+00	5.20E-02	5.32E-03	0.05
Ubiquinone and other terpenoid-quinone biosynthesis	36	1	2.74E-03	5.90E+00	5.20E-02	5.32E-03	0.00
Phenylalanine metabolism	45	2	4.04E-03	5.51E+00	6.87E-02	7.44E-03	0.12
Glutathione metabolism	38	3	1.77E-02	4.04E+00	2.83E-01	3.09E-02	0.01
Lysine degradation	47	2	2.81E-02	3.57E+00	4.22E-01	4.68E-02	0.15
Porphyrin and chlorophyll metabolism	104	3	3.37E-02	3.39E+00	4.71E-01	5.36E-02	0.00
beta-Alanine metabolism	28	5	1.86E-01	1.68E+00	1.00E+00	2.81E-01	0.28
Primary bile acids biosynthesis	47	2	1.93E-01	1.65E+00	1.00E+00	2.81E-01	0.02
Propanoate metabolism	35	1	2.16E-01	1.53E+00	1.00E+00	3.03E-01	0.09
Glycine. serine and threonine metabolism	48	7	2.59E-01	1.35E+00	1.00E+00	3.48E-01	0.44

Thiamine metabolism	24	2	3.10E-01	1.17E+00	1.00E+00	4.02E-01	0.00
Sphingolipid metabolism	25	1	3.66E-01	1.01E+00	1.00E+00	4.41E-01	0.00
Sulfur metabolism	18	1	3.66E-01	1.01E+00	1.00E+00	4.41E-01	0.00
Cyanoamino acid metabolism	16	4	3.84E-01	9.58E-01	1.00E+00	4.47E-01	0.00
Methane metabolism	34	2	3.99E-01	9.19E-01	1.00E+00	4.50E-01	0.02
Taurine and hypotaurine metabolism	20	2	4.57E-01	7.84E-01	1.00E+00	4.99E-01	0.36
Cysteine and methionine metabolism	56	5	6.22E-01	4.75E-01	1.00E+00	6.57E-0.1	0.18
Selenoamino acid metabolism	22	1	6.38E-01	4.49E-01	1.00E+00	6.57E-01	0.00
Valine. leucine and isoleucine biosynthesis	27	1	8.02E-01	2.21E-01	1.00E+00	8.02E-01	0.00

Table S3. The most important impact of pathway analysis with MetPA from serum endogenous metabolites. Comparison metabolites from serum of lean controls with post-OAGB patients.

	Total Cmpd	Hits	Raw p	-log(p)	Holm adjust	FDR	Impact	Pivotal amino acid	P value
Biotin metabolism	11	1	2.82E-11	2.43E+01	9.86E-10	5.12E-10	0.00	L-Lysine	2.81E-11
Lysine biosynthesis	32	2	2.92E-11	2.43E+01	9.94E-10	5.12E-10	0.10	L-Lysine	2.81E-11
beta-Alanine metabolism	28	5	6.83E-05	9.59E+00	2.26E-03	7.97E-04	0.28	GABA	2.16e-10
Tryptophan metabolism	79	1	3.45E-04	7.97E+00	1.10E-02	3.02E-03	0.11	L-Tryptophan	0.0003
Phenylalanine. tyrosine and tryptophan biosynthesis	27	3	6.18E-04	7.39E+00	1.92E-02	4.32E-03	0.01	L-Phenylalanine	0.0001
Phenylalanine metabolism	45	2	1.44E-03	6.55E+00	4.31E-02	8.37E-03	0.12	L-Phenylalanine	0.0001
Lysine degradation	47	2	1.75E-03	6.35E+00	5.08E-02	8.76E-03	0.15		
Butanoate metabolism	40	2	2.85E-03	5.86E+00	7.97E-02	1.21E-02	0.01		
D-Arginine and D-ornithine metabolism	8	2	3.11E-03	5.77E+00	8.41E-02	1.12E-02	0.00		
Tyrosine metabolism	76	1	7.80E-03	4.85E+00	2.03E-01	2.48E-02	0.05		
Ubiquinone and other terpenoid-quinone biosynthesis	36	1	7.80E-03	4.85E+00	2.03E-01	2.48E-02	0.00		
Valine. leucine and isoleucine biosynthesis	27	1	9.12E-03	4.70E+00	2.19E-01	2.66E-02	0.00		
Histidine metabolism	44	5	2.75E-02	3.59E+00	6.33E-01	7.40E-02	0.15		
Aminoacyl-tRNA biosynthesis	75	16	3.38E-02	3.39E+00	7.44E-01	8.46E-02	0.23		

Arginine and proline metabolism	77	9	1.52E-01	1.88E+00	1.00E+00	3.49E-01	0.44
Taurine and hypotaurine metabolism	20	2	1.71E-01	1.77E+00	1.00E+00	3.49E-01	0.36
Nicotinate and nicotinamide metabolism	44	1	1.72E-01	7.76E+00	1.00E+00	3.49E-01	0.00
Nitrogen metabolism	39	11	1.80E-01	1.72E+00	1.00E+00	3.49E-01	0.01
Alanine. aspartate and glutamate metabolism	24	6	2.05E-01	1.59E+00	1.00E+00	3.56E-01	0.85
D-Glutamine and D-glutamate metabolism	11	2	2.09E-01	1.56E+00	1.00E+00	3.56E-01	0.14
Pantothenate and CoA biosynthesis	27	2	2.14E-01	1.54E+00	1.00E+00	3.56E-01	0.07
Pyrimidine metabolism	60	2	2.41E-01	1.42E+00	1.00E+00	3.66E-01	0.00
Selenoamino acid metabolism	22	1	2.47E-01	1.40E+00	1.00E+00	3.66E-01	0.00
Cysteine and methionine metabolism	56	5	2.51E-01	1.38E+00	1.00E+00	3.66E-01	0.18
Primary bile acid biosynthesis	47	2	3.13E-01	1.16E+00	1.00E+00	4.38E-01	0.02
Glutathione metabolism	38	3	3.54E-01	1.04E+00	1.00E+00	4.76E-01	0.01
Porphyrin and chlorophyll metabolism	104	3	3.98E-01	9.22E-01	1.00E+00	5.15E-01	0.00
Purine metabolism	92	2	4.27E-01	8.51E-01	1.00E+00	5.34E-01	0.00
Glycine. serine and threonine metabolism	48	7	4.56E-01	7.85E-01	1.00E+00	5.50E-01	0.44
Propanoate metabolism	35	1	5.14E-01	6.61E-01	1.00E+00	6.02E-01	0.09
Thiamine metabolism	24	2	5.79E-01	5.46E-01	1.00E+00	6.54E-01	0.00

Sphingolipid metabolism	25	1	8.40E-01	1.74E-01	1.00E+00	8.91E-01	0.00
Sulfur metabolism	18	1	8.40E-01	1.74E-01	1.00E+00	8.91E-01	0.00
Cyanoamino acid metabolism	16	4	8.82E-01	1.25E-01	1.00E+00	9.08E-01	0.00
Methane metabolism	34	2	9.25E-01	7.77E-02	1.00E+00	9.25E-01	0.02

Table S4. The most important impact of pathway analysis with MetPA from serum endogenous metabolites. Comparison metabolites from serum of pre-OAGB with post-OAGB patients.

	Total Cmpd	Hits	Raw p	-log(p)	Holm adjust	FDR	Impact	Pivotal amino acid	P value
Histidine metabolism	44	5	5.33E-07	1.44E+01	1.87E-05	1.03E-05	0.15	L-Glutamate	4.60E-07
Butanoate metabolism	40	2	5.86E-07	1.43E+01	1.99E-05	1.03E-05	0.01	L-Glutamate	4.60E-07
Arginine and proline metabolism	77	9	4.72E-06	1.23E+01	1.56E-04	2.51E-05	0.44	G-Glutamate; L-Glutamine	4.60E-07; 0.0001
D-Glutamine and D-glutamate metabolism	11	2	6.75E-06	1.19E+01	2.16E-04	5.91E-05	0.14	L-Glutamate	4.60E-07
Nitrogen metabolism	39	11	3.07E-05	1.04E+01	9.52E-04	2.15E-04	0.01		
Pyrimidine metabolism	60	2	1.95E-04	8.75E+00	5.84E-03	1.14E-03	0.00		
Purine metabolism	92	2	5.41E-04	7.52E+00	1.57E-02	2.70E-03	0.00		
beta-Alanine metabolism	28	5	7.94E-04	7.14E+00	2.22E-02	3.07E-03	0.28		
Lysine biosynthesis	32	2	8.23E-04	7.10E+00	2.22E-02	3.07E-03	0.10		

Biotin metabolism	11	1	8.77E-04	7.04E+00	2.28E-02	3.07E-03	0.00
Alanine. aspartate and glutamate metabolism	24	6	1.13E-03	6.79E+00	2.82E-02	3.50E-03	0.85
Valine. leucine and isoleucine biosynthesis	27	1	1.20E-03	6.72E+00	2.88E-02	3.50E-03	0.00
Aminoacyl-tRNA biosynthesis	75	16	1.41E-03	6.56E+00	3.25E-02	3.80E-03	0.023
Porphyrin and chlorophyll metabolism	104	3	2.42E-03	6.02E+00	5.32E-02	6.05E-03	0.00
Glutathione metabolism	38	3	3.25E-03	5.73E+00	6.82E-02	7.58E-03	0.01
Tryptophan metabolism	79	1	8.08E-03	4.82E+00	1.02E-01	1.77E-02	0.11
Nicotinate and nicotinamide metabolism	44	1	3.60E-02	3.32E+00	6.85E-01	7.42E-02	0.00
Pantothenate and CoA biosynthesis	27	2	4.40E-02	3.12E+00	7.92E-01	8.56E-02	0.07
D-Arginine and D-ornithine metabolism	8	2	1.19E-01	2.13E+00	1.00E+00	2.18E-01	0.00
Lysine degradation	47	2	1.27E-01	2.06E+00	1.00E+00	2.23E-01	0.15
Phenylalanine. tyrosine and tryptophan biosynthesis	27	3	1.74E-01	1.75E+00	1.00E+00	2.89E-01	0.01
Glycine. serine and threonine metabolism	48	7	1.89E-01	1.66E+00	1.00E+00	3.01E-01	0.44
Sphingolipid metabolism	25	1	2.30E-01	1.47E+00	1.00E+00	3.35E-01	0.00
Sulfur metabolism	18	1	2.30E-01	1.47E+00	1.00E+00	3.35E-01	0.00
Cyanoamino acid metabolism	16	4	2.78E-01	1.28E+00	1.00E+00	3.82E-01	0.00
Methane metabolism	34	2	2.86E-01	1.25E+00	1.00E+00	3.82E-01	0.02

Thiamine metabolism	24	2	2.99E-01	1.21E+00	1.00E+00	3.82E-01	0.00
Primary bile acid biosynthesis	47	2	3.06E-01	1.18E+00	1.00E+00	3.82E-01	0.02
Propanoate metabolism	35	1	4.08E-01	8.96E-01	1.00E+00	4.93E-01	0.09
Cysteine and methionine metabolism	56	5	4.98E-01	6.98E-01	1.00E+00	5.81E-01	0.18
Selenoamino acid metabolism	22	1	5.14E-01	6.65E-01	1.00E+00	5.81E-01	0.00
Taurine and hypotaurine metabolism	20	2	5.32E-01	6.31E-01	1.00E+00	5.82E-01	0.36
Phenylalanine metabolism	45	2	5.75E-01	5.53E-01	1.00E+00	6.10E-01	0.12
Tyrosine metabolism	76	1	9.69E-01	3.17E-01	1.00E+00	9.69E-01	0.05
Ubiquinone and other terpenoid-quinone biosynthesis	36	1	9.69E-01	3.17E-01	1.00E+00	9.69E-01	0.00
